# Embracing complexity to challenge stigma: a qualitative analysis of representations of abortion in a Polish storytelling initiative

**DOI:** 10.1080/26410397.2025.2535835

**Published:** 2025-08-15

**Authors:** Alina Paczesna

**Affiliations:** MSC Student, London School of Economics and Political Science, London, UK. *Correspondence*: a.paczesna@lse.ac.uk

**Keywords:** abortion, abortion stigma, abortion storytelling, pro-choice, Poland

## Abstract

Testimonies of abortion experiences are largely silenced in many contexts around the world, including Poland, and stigma affects how abortion is represented. Pro-choice initiatives, which support abortion rights, aim to challenge stigma through the sharing of personal abortion experiences (i.e. abortion storytelling). However, these initiatives may simultaneously construct normative hierarchies of abortion and stigmatise abortion stories which do not fit context-specific, politicised norms. This study draws on data purposefully sampled from a pro-choice abortion storytelling initiative in Poland, conducted between 2020 and 2021, and organised by the Abortion Dream Team (ADT; Aborcyjny Dream Team), an influential Polish organisation campaigning for abortion access and rights. By conducting a qualitative document analysis of 73 first-person abortion stories shared in this initiative, I explored how abortion was represented, and whether, and how, these representations challenged abortion stigma and its manifestation in normative hierarchies of abortion. Using reflexive thematic analysis, I identified four main themes. My findings show that abortion is represented as a valid decision, both a positive and challenging experience, and an embodied process. The stories shared in the ADT storytelling initiative challenge stigma and deconstruct normative hierarchies of abortion by representing abortion as a complex experience imbued with multiple, and often contradictory, meanings. This study highlights the existence of positive and non-stigmatising representations of abortion in Poland and shows that abortion storytelling can challenge dominant narratives around abortion. These findings therefore have broader significance, as they suggest that abortion storytelling may serve as an effective tool to destigmatise abortion and advance abortion rights. DOI:10.1080/26410397.2025.2535835

## Plain language summary

In many contexts around the world, including Poland, abortion is stigmatised. Stigma shapes how we think about and understand abortion, as it contributes to constructing abortion as something negative and undesirable. This makes it difficult for people to talk about their abortion experiences freely. But there are initiatives that challenge stigmatising and negative representations of abortion exist. This helps reshape how abortion is portrayed and made sense of. However, these initiatives may still privilege or silence particular abortion stories, creating hierarchies of abortion narratives. This study analysed 73 abortion stories published in a Polish abortion storytelling initiative. This initiative was organised by the Abortion Dream Team (ADT; Aborcyjny Dream Team). ADT campaigns for abortion rights and access, and aims to challenge the silence around abortion. I explored how abortion was represented in these stories and how these representations challenged abortion stigma and related hierarchies of abortion. My findings show that abortion was represented as a valid decision, as an experience that can be both positive and challenging, and as a bodily process. The stories represent abortion as a complex experience which has multiple, often contradictory meanings. This challenges stigma and hierarchies of abortion. This study highlights that positive and non-stigmatising representations of abortion exist in Poland. This is important as abortion stigma constitutes a barrier to reproductive freedom.

## Résumé

Les témoignages sur l’avortement sont largement passés sous silence dans de nombreux contextes à travers le monde, notamment en Pologne, et la stigmatisation influence la représentation de l’avortement. Les initiatives pro-choix, qui soutiennent le droit à l’avortement, vise à lutter contre la stigmatisation en partageant des expériences personnelles de l’avortement (c’est-à-dire des récits sur l’avortement). Néanmoins, ces initiatives peuvent simultanément construire des hiérarchies normatives de l’avortement et stigmatiser les récits d’avortement qui ne correspondent pas aux normes politisées et spécifiques au contexte. Cette étude s’appuie sur les données d’une initiative pro-choix de récits d’avortement en Pologne avec échantillon sélectionné par choix raisonné entre 2020 et 2021; on doit cette initiative à la Dream Team de l’avortement (ADT; Aborcyjny Dream Team), une organisation polonaise influente militant pour l’accès et le droit à l’avortement. En menant une analyse documentaire qualitative de soixante-treize récits d’avortement, partagés à la première personne dans le cadre de cette initiative, j’ai étudié la manière dont l’avortement était représenté, ainsi que si, et comment, ces représentations remettaient en question la stigmatisation de l’avortement et sa manifestation dans les hiérarchies normatives de l‘avortement. À l’aide d’une analyse thématique réflexive, j’ai identifié quatre thèmes principaux. Mes résultats montrent que l’avortement est présenté comme une décision valable, une expérience à la fois positive et difficile, et un processus incarné. Les récits partagés dans le cadre de l’initiative d’ADT remettent donc en question la stigmatisation et déconstruisent les hiérarchies normatives de l’avortement en le présentant comme une expérience complexe, imprégnée de significations multiples et souvent contradictoires. Cette étude met en évidence l’existence de représentations positives et non stigmatisantes de l’avortement en Pologne, et montre que les récits sur l’avortement peuvent remettre en question les discours dominants autour de l’avortement. Ces résultats ont donc une portée plus large, puisqu’ils suggèrent que les récits sur l’avortement peuvent constituer un outil efficace pour déstigmatiser l’avortement et faire progresser le droit à l’avortement.

## Resumen

Los testimonios sobre experiencias de aborto son principalmente silenciados en muchos contextos del mundo, entre ellos Polonia, y el estigma afecta la manera en que se representa el aborto. Iniciativas por la libre elección, que apoyan el derecho al aborto, procuran afrontar el estigma al compartir experiencias de aborto personales (ej., narración de historias de aborto). Sin embargo, estas iniciativas podrían construir simultáneamente jerarquías normativas del aborto y estigmatizar las historias de aborto que no encajan en las normas politizadas específicas del contexto. Este estudio está basado en datos de una iniciativa de narración de historias de aborto en Polonia, a favor de la libre elección y con un muestreo intencional entre 2020 y 2021, organizada por el *Abortion Dream Team* (ADT; Aborcyjny Dream Team), influyente organización polaca que hace campaña por el acceso y el derecho al aborto. Al realizar un análisis documental cualitativo de setenta y tres historias de aborto en primera persona compartidas durante esta iniciativa, exploré cómo se representaba el aborto, al igual que si, y cómo, esas representaciones afrontaban el estigma del aborto y su manifestación en las jerarquías normativas del aborto. Utilizando análisis reflexivo temático, identifiqué cuatro temas principales. Mis hallazgos muestran que el aborto es representado como una decisión válida, como una experiencia tanto positiva como difícil, y como un proceso incorporado. Por tanto, las historias compartidas en la iniciativa del ADT de narración de historias afrontan el estigma y deconstruyen las jerarquías normativas del aborto al representar el aborto como una experiencia compleja impregnada de múltiples significados, a menudo contradictorios. Este estudio destaca la existencia de representaciones positivas y no estigmatizantes del aborto en Polonia, y muestra que la narración de historias de aborto puede cuestionar las narrativas dominantes sobre el aborto. Por consiguiente, estos hallazgos tienen mayor importancia, ya que indican que la narración de historias de aborto podría ser una herramienta eficaz para desestigmatizar el aborto y promover el derecho al aborto.

## Introduction

In 2016, Polish anti-choice actors proposed the “Stop Abortion” bill which, if passed, would criminalise abortion in Poland entirely, thereby challenging the previous 1993 abortion law.^1^ The 1993 legislation was already restrictive, permitting abortion only in cases of rape, fetal anomaly or threat to the pregnant person’s life or health; however, abortion-seekers were not criminalised, even when abortions were self-managed.^1^ Though the 2016 Women’s Strikes pressured the right-wing government into rejecting the “Stop Abortion” bill,^2^ in 2020 the ruling party-aligned Constitutional Tribunal declared abortion due to fetal anomaly unconstitutional, outlawing 96% of state-sanctioned abortions.^3^ Despite these restrictions, around 100,000 Poles have abortions each year,^4^ as people circumvent the law by travelling abroad, using “underground” clinics,^5,6^ or using abortion pills to terminate their pregnancies at home.^1^ Though abortion is common, testimonies of people’s experiences have largely been absent from public debate in Poland^5^ as abortion has been both “criminalised and stigmatised” (p.99).^7^ However, recent pro-choice initiatives mobilise abortion storytelling to challenge stigma. As this study shows, they do so successfully by portraying abortion as a complex and multifaced experience, and thereby disrupting the rigid and monolithic narratives that characterise abortion stigma.

Abortion stigma can be defined as “a negative attitude ascribed to women who seek to terminate a pregnancy that marks them […] as inferior to ideals of womanhood”, including inevitable motherhood, nurture and procreative sexuality.^8^ Such stigma manifests on various levels.^8^ On an individual level, abortion stigma can be internalised and produce guilt and shame in those who have abortions, while on a community level, it may contribute to ostracising abortion-seekers.^8^ Stigma also impacts how abortion is understood, framed and represented.^8^ Overall, abortion stigma works to differentiate women who have had abortions from women who have not.^9^ While researchers have extensively explored how women experience and navigate community- and individual-level stigma,^10^ it is also important to consider how abortion is represented, and whether various representations challenge or reinforce stigma. This informs the focus of my study.

Abortion stigma can be understood as a violation of reproductive rights, as it fosters discriminatory attitudes and creates barriers that restrict individuals’ ability to access safe and legal abortion care.^10^ In Poland, abortion stigma was reinforced by the commercialisation and privatisation of abortion that resulted from the restrictive 1993 legislation: in the absence of state provision, women turned to private abortion providers, abortion went “underground” and was therefore less publicly visible.^7^ This lack of visibility, reinforced by the fact that women increasingly sought clandestine, private sector abortions, contributed to further exceptionalising abortion.^7^ Further, while the Catholic Church had been publicly opposing abortion throughout the twentieth century, in the 1980s and 1990s Catholic discourses became increasingly influential, and further stigmatised abortion by constructing it as “morally wrong” and “unacceptable to speak of”, which discouraged women from sharing their experiences.^1,7^ In Poland, abortion stigma therefore manifests itself in the fact that abortions happen but are not talked about, and therefore constitute a “public secret” (p.17).^1^

The silencing of abortion testimonies undermines how common abortions are and contributes to constructing abortion as exceptional.^8,11^ Stigma therefore shapes how abortion is made sense of and understood – not as a normal, common experience, but rather as an abnormal event. However, discourses of abortion exceptionalism do not affect all abortions equally, but stigmatise or legitimise different abortions “depending on the circumstances of pregnancy” (p.182).^12^ For example, abortions that do not stem from medical necessity are often perceived as less legitimate than those that do.^13^ The perceived legitimacy of abortion is further influenced by performing socially expected emotions^14^ – for example, women should perform grief for their fetuses, which constructs abortion as a negative experience.^15^ Altogether, these norms construct “hierarch[ies] of abortion narratives regarding which kinds of abortions are socially acceptable and worthy of talking about” (p.2).^16^ Though these hierarchies may be constructed around a variety of issues, including women’s reasons for and emotional responses to abortion, or the gestational age at which abortions were obtained, in Poland hierarchies centre around reasons and emotions; I explore this in more detail further below. Overall, normative hierarchies (re)produce stigma by constructing rigid norms around which abortions are acceptable and which abortion stories can be told. This then results in the othering of women who fail to conform to these norms.^9,17^ However, as stigma “is contingent and contested”, it is only “one of many processes through which abortion is made intelligible” (p.6).^10^ It therefore can be, and is, challenged through positive and non-stigmatising representations, such as those de-centring the fetus and centring the woman’s needs and wants.^17^ The “othering” which creates hierarchies of abortion may, however, also be perpetuated in women’s abortion narratives, including those shared in pro-choice storytelling projects.^18,19^

Pro-choice projects often construct their own norms around “acceptable” abortions, even when these differ from mainstream norms, which highlight the dynamic character of normative hierarchies of abortion.^20,21^ Existing research on pro-choice storytelling, conducted in the United States and Ireland, is split, however, on how these hierarchies are constituted. Some studies show that positive, “unapologetic” narratives dominate some campaigns.^22^ This creates “expectations for what an abortion should look and feel like”, for example, with “relief, empowerment, and low attachment to the pregnancy” (p.473)^23^ being expected, potentially ostracising those who feel otherwise.^20^ Other research shows that stories told in pro-choice spaces are apologetic, with women rationalising their decisions, often through comparison to others who are constructed as illegitimate abortion-havers, or by positioning abortion as a regrettable and negative event.^15,24,25^ Brought together, this scholarship highlights that even when available narratives are expanded beyond negative ones, pro-choice abortion stories simultaneously construct their own diverse and context-specific norms and hierarchies.^15,22,23^ Storytelling projects which reproduce normative hierarchies of abortion are therefore characterised by a “narrative” and “thematic” rigidity.^21,26^ As normative hierarchies reproduce stigma by restricting which stories and experiences can be talked about, efforts to challenge stigma are inherently limited in such initiatives. However, the above research focuses on the United States and Ireland, and may not be directly generalisable onto Poland.

In Poland, pro-choice counter-narratives tend to reproduce the idea that abortion should be “safe, legal and rare” (p.16).^11^ Emphasising that abortion should be rare “suggests that […] there are some conditions for which abortions should and should not occur” (p.69).^27^ The narratives put forward during the 2016 protests arguably reproduced normative hierarchies of abortion. Specifically, unapologetic and positive abortion stories were downplayed during protests, while abortions that conformed to hegemonic “familial and maternal discourses”, for example those that framed abortions as acts of care for existing or future children, were privileged (p.15).^11^ However, protesters simultaneously challenged Catholic discourses of womanhood and motherhood, creating space for different norms and drawing attention to women’s lived experiences.^28^ New “abortion self-help” initiatives, focusing on abortion provision, were also created.^28,29^ This includes organisations like the Abortion Dream Team (ADT; Aborcyjny Dream Team) which, apart from facilitating abortions, aims to challenge exceptionalism and secrecy by talking about abortion as a common and normal event, and creating space for the articulation of various abortion experiences.^30^ Pro-choice activism radicalised further after the 2020 legislative changes, when the Church’s authority was explicitly renounced at protests, contributing to a further shift beyond Catholic, stigmatising discourses of abortion.^28,30^ More women than before began to publicly share their abortion experiences, including on purpose-made online storytelling platforms.^28,30^ These recent storytelling initiatives have not been studied; it is therefore unclear whether and, if so, how these initiatives reproduce normative hierarchies of abortion. However, online abortion storytelling projects provide crucial insights into narratives dominant in pro-choice movements – individual stories show how people construct their experiences, while the narratives altogether highlight wider norms.^26,31^

The aim of this study is to examine how abortion is represented in stories shared in a Polish online abortion storytelling initiative organised by ADT. Specifically, this research asks whether, and how, representations of abortion in these stories contribute to challenging abortion stigma, and what these stories tell us about normative hierarchies of abortion in Polish pro-choice spaces. As stigma and pro-choice norms are both context-specific and historically contingent,^10^ abortion stories shared in post-2020 Polish storytelling initiatives can, and do, differ from stories shared in anglophone initiatives.

## Methodology

### Research design

This study is a qualitative document analysis of first-person abortion stories shared in a Polish online abortion storytelling initiative, organised by ADT. Qualitative document analysis allowed me to investigate how women’s stories “amount to a collective narrative” (p.82) of abortion that is constructed through sharing individual experiences.^32^ As I could not probe story authors to get detailed insights about what they experienced, qualitative document analysis was unsuitable for studying experiences of abortion. However, it enabled an inductive study into narratives produced in a particular historical moment,^33^ allowing for the exploration of representations of abortion in post-2020 Poland. My approach to studying representations broadly aligns with Hall’s^34^ interpretation – in exploring representations of abortions, I examine what meanings about abortion are produced.

### Sampling and data collection

Purposive sampling^33^ was used to select an online storytelling project initiated by ADT, and titled “I had an abortion” (“Miałam aborcję”).^35^ This initiative was selected because ADT is a major Polish activist organisation that campaigns for abortion rights, facilitates abortion provision and educates about abortion in Poland.^30,36^ ADT is also a significant actor in the European abortion landscape, and actively collaborates with international and transnational organisations working towards abortion access, including Abortion Without Borders, among others.^36^ Furthermore, ADT explicitly aims to challenge abortion stigma through storytelling,^36^ which enables an exploration of narratives in a “pro-choice” space. ADT gave no prompts for story submission, highlighted that all experiences matter, and informed potential contributors that their stories would be shared on the ADT website and that only hate-speech and misinformation would not be published.^35^ However, it potentially could control the stories published, and their content, which could influence the narratives present across the dataset. The stories shared in the ADT initiative therefore cannot be understood to be representative of all women’s abortion experiences, but do provide insight into narratives present in this pro-choice space.

Further selection criteria were developed for individual stories. Stories must have been published between 22.10.2020 and 22.10.2021, to examine storytelling in the first year after the 2020 abortion ban. Although it became legally binding on 27.01.2021, the publication of the ruling on 22.10.2020 initiated large-scale protests,^28^ which could have influenced abortion narratives.^21^ As it could not be determined when the abortions described in the stories happened, I did not sample for stories describing abortions that happened in a particular historical moment. However, as my analysis concerns representations rather than experiences of abortion, the exact timings of the abortions themselves are not analytically relevant. Only stories written in first person where the contributors were describing their abortions are included, as I focus on “narratives of women who have had abortions” (p.226–7).^37^

Data collection was fully completed on 17.05.2023, when all stories were copied verbatim from the website, checked against the sampling criteria, and imported into NVivo.

### Sample

Seventy-three stories met all sampling criteria and were included. They were published between 21.03.2021 and 19.10.2021. The stories varied in length, ranging from 68 to 1130 words, with an average word count of 335 words. Demographic information about contributors (i.e. story authors) was not collected by ADT as part of the story submission process and was therefore generally unavailable. However, it could be inferred from the stories that most contributors were in heterosexual relationships and lived in Poland. Many shared that they had children. Most contributors stated that they had one abortion, while some shared that they had multiple.

### Data analysis

All data analysis was carried out by the author. I used reflexive thematic analysis (RTA) to analyse the data, as it allowed me to identify patterns of meaning around representations of abortion, whilst simultaneously “highlight[ing] the researcher’s active role in knowledge production” (p.1393)^38^ and in producing “interpretive stories about the data” (p.594).^39,40^ Though RTA is reiterative, it involves six main stages.^40,41^ After initial familiarisation, I coded the data inductively, i.e. the analysis was “‘grounded in’ the data” (p.331)^40^ Inductive coding allowed for a detailed exploration of data collected using non-reactive methods where probing to explore specific themes in-depth was unfeasible.^42^ Data were coded using NVivo software (version 1.7.1). Two rounds of coding were sufficient to capture all important meanings. I then grouped similar codes together and identified initial themes, or “patterns of shared meaning, cohering around a central concept” (p.331).^40^ I then re-read coded extracts for each theme and amended themes where necessary, ensuring they reflect meanings in the data. Themes changed multiple times, until I decided on the scope of each.^38,41^

### Translation

The dataset was in Polish, and I moved between English and Polish throughout coding and analysis, using the language which enabled me to best capture underlying meanings.^43^ I translated all cited data excerpts myself and made context-specific translation choices to avoid losing any crucial meaning.^44^

### Ethics

This study received ethical approval through the University of Edinburgh’s School of Social and Political Science ethics review on 3rd April 2023. This research was further informed by a feminist ethics of care.^45^ Therefore, though protecting contributors’ anonymity was formally unnecessary as the data were public, as contributors shared experiences which are arguably sensitive in “the social context within which the research [was] conducted” (p.5)^46^ and as the data were not produced for research purposes, contributors’ names are not used throughout this report. All quotes are reported without any identifying information.

### Reflexivity and positionality

I am a cisgender Polish woman, and Polish law has constrained my reproductive freedom, a constraint which I share with my contributors. However, this does not imply that my perspectives and politics converge with either individual contributors or the wider storytelling initiative,^47^ so I have critically engaged with my assumptions throughout to ensure that contributors’ perspectives are truthfully represented.^48^

## Results

Though the multiplicity of narratives in the stories means that not everything could be discussed in this article, four main themes, prevalent across the dataset, were identified: abortion was represented as a valid decision, a positive experience, a challenging experience, and an embodied process.

### Abortion decisions are valid decisions

Many contributors constructed abortion as a valid reproductive decision that pregnant women should be able to make. When talking about abortion decisions in the abstract, many explicitly rendered abortion an acceptable choice regardless of reason:

“*Remember that this is your choice and you will have to live with this decision for the rest of your life. […] Every reason is good – there are no inadequate reasons – every [reason] is good, without exception, if only you feel that this is the right path for you.*”

Other contributors also explicitly rejected the notion that some reasons for abortion are “inadequate”, thereby imbuing every reason for abortion with equal value. In such stories, what makes an acceptable abortion decision is whether “this is the right path for you” – pregnant women’s subjectivities, needs and desires are foregrounded, and abortion is constructed as a decision women should be able to make if they wish to. This also implicitly rejects other people’s authority to judge their decisions.

Abortion was not only discussed in the abstract – it was a lived experience for all contributors and the stories focused on women’s own experiences of abortion. Most contributors gave diverse reasons for why they terminated their pregnancies. When explaining their abortion decisions, some women highlighted the adverse circumstances they found themselves in:

“*[…] my financial situation is unstable, I haven’t finished university, I still live with my parents. I’m not saying that I wouldn’t be able to cope, because I definitely would – but I wouldn’t want to raise a child like that – barely making ends meet, thinking about how to combine my studies, work and early motherhood.*”

What helps explain the abortion decision here are the conditions the contributor would become a mother in, which she understands to be inadequate and undesirable. The above excerpt also hints at notions of children’s wellbeing, which others also drew on:

“*I made the decision that there will be no child, […] and I’m not hurting anyone but rather doing a favour, because I cannot afford to provide them with the right conditions – in 9 months I won’t be able to either.*”

In such stories, abortion becomes a correct choice through the assertion that it would benefit the potential child by sparing it from a life without “the right” resources and care. Such explanations were also present in accounts of abortion due to fetal anomaly, where abortion constituted a means of preventing the (potential) child’s suffering.

These rationalisations existed alongside other, more transgressive narratives. Many women foregrounded their needs and wants when discussing their abortions:

“*I didn’t want that pregnancy, that child. It’s not even that I didn’t have the capacity and conditions for a second child, I just simply did not want it.*”

“*I’m 31 years old and I’m currently defining and achieving my life goals. Currently a child is not one of them, and I don’t see myself as a mother, at least not in the near future.*”

These testimonies challenge the focus on economic reasons and suitable conditions for childbearing. Instead, here both contributors draw on personal plans and desires (i.e. not wanting more children or wanting to achieve “life goals”), and understand them as valid reasons for abortions. However, they do not frame this as decisions made to benefit their (future) children, but themselves. Other women also explained their decisions by explicitly rejecting motherhood:

“*I’m 31 years old, I have a husband, family, friends. I have savings and a good job. But I don’t want to be a mother. I just don’t.*”

### *“*I cried tears of happiness”: abortion as a positive experience

Abortion was not only constructed as a valid decision, but was also represented as a positive experience. Contributors established abortion as explicitly positive by discussing their emotions, throughout and after their abortions, and the overall positive impact of abortion:

*“I am free and feel great!”*

*“[After the abortion] I was left with a feeling of relief […]. I felt that I am starting a new life.”*

In these excerpts, emotions such as “relief” and feeling “free” and “great” are reported directly and without qualification, thereby constructing abortion as something that constitutes a positive event. Through framing abortion as a life-giving decision (“I am starting a new life”), this contributor further foregrounds the positive impact the abortion had on her life. This framing relies on acknowledging that a pregnancy may constitute a negative and undesirable event in someone’s life when it is unwanted; abortion therefore becomes a positive event as it ends this unwanted state.

Beyond only implicitly suggesting that their (unwanted) pregnancies were undesirable, some contributors explicitly juxtaposed their poor physical and emotional condition during the pregnancy with the improved sense of wellbeing they regained after the abortion:

*“I feel great, I regained my positive attitude and joy of everyday life, the pregnancy symptoms stopped bothering me, I can have a proper meal again without being afraid that I’ll throw up, it’s like before [the pregnancy] – or even better, because I appreciate it now.”*

Here, pregnancy is represented as a time when the contributor felt unwell, both physically and emotionally. The contributor foregrounds her subjective experience to highlight that pregnancy can be both undesirable and unpleasant, and that abortion can therefore constitute a positive and life-giving decision as it allows women to regain the “joy” and wellbeing they felt before becoming pregnant.

Contributors further constructed abortion as a positive experience by imbuing the abortion process with positive emotions:

*“When I took the first pill I felt relief. No more fear, all of a sudden. The following day when I took the next pills and started bleeding I cried tears of happiness.”*

Abortion is often presented as an exceptional, traumatising and “horrible” medical procedure.^17,26^ However, here the moment of initiating a self-managed abortion is presented as a positive one. The abortion itself is marked as a happy moment, arguably because it constitutes the process of ending the (unwanted) pregnancy that was causing the contributor’s distress. However, not all contributors’ pregnancies were unwanted.

### “It pains me that l had to make such a difficult decision”: abortion as a challenging experience

Despite the overwhelming positivity around abortion outlined above, a minority of contributors experienced their abortions as difficult, thereby constructing abortion as challenging. Importantly, these abortions were still understood to be legitimate decisions, as contributors did not construct abortion as wrong, but highlighted complexities of abortion decisions and experiences that compound to make some abortions challenging. For example, some contributors were ambivalent and conflicted about their pregnancies and abortions:

*“I was not ready for a child […]. I have always felt that this is not my path. Motherhood is not for me […]. Despite this, when I saw the two lines on the pregnancy test […] I began to hesitate, I started to wonder whether I could manage after all. With my boyfriend, we made the decision to terminate because both of us are not ready [for a child]. At first I was a bit sad that this happened, I never wanted to go through it, but it happened. My head was a complete mess.”*

While it is unclear whether it was the pregnancy or the abortion that made the contributor “a bit sad” that it “happened”, this extract captures the ambivalence she felt about the situation she found herself in. Arguably, she did not want to have an abortion, but neither did she want to remain pregnant. This disrupts the positivity outlined above by showing that the distinction between wanted and unwanted pregnancies is not clear-cut, and that abortion decisions are complex, also because people have to make choices they would rather have avoided. Abortion is not constructed as wrong, but as a complex experience – something you may not want to have to decide about, but simultaneously a worthwhile and correct choice.

However, not all contributors were ambivalent about their pregnancies; some terminated wanted pregnancies, which was emotionally demanding. This was true of all abortions following a fetal congenital malformation diagnosis:

“Ever since I made that decision I cried every day, I also changed that decision in my mind every day. It was definitely the most difficult time for us both. I knew that this was also difficult for my husband, but we had to act. I found Ciocia Czesia* *and now my little one no longer greets me with a kick in the morning.”*

This abortion was difficult because of the particularities of the situation this woman was in, and the fact that her pregnancy was wanted, but could not be carried to term. This contributor expresses emotional attachment to her pregnancy, and arguably also her fetus. Her story also highlights how some choices are constrained; other abortions were also framed as challenging as they stemmed from a perceived lack of choice:

*“It pains me that I had to make such a difficult decision, but I couldn’t do anything else. I regret that we live in a country where, not to even mention the availability of such pills [abortion pills], I, a 22-year-old, cannot afford to have a child. It’s terrible that a woman has to suffer such pain because she wants to [have a child], but can’t.”*

Here, the difficulty and “pain” that the contributor experienced is not linked to the abortion as such, but rather to how her decision was shaped by the fact that she “cannot afford to have a child”. This extract critiques the “country where […] I […] cannot afford to have a child” – the contributor understands the state to be responsible, at least partially, for producing these structural economic concerns, which resulted in her abortion being experienced as challenging. Just as in fetal anomaly cases, continuing the pregnancy here is a “non-option”, this time due to economic constraints.^49,50^ Abortion therefore becomes a necessary and challenging decision.

### Cramps, blood and pain: abortion as an embodied process

Abortion was further represented as a profoundly physical and embodied experience. In recent years, interview-based research across a variety of contexts has examined the embodied experiences of abortion, including abortion-related pain.^51–55^ However, the embodiment of abortion was rarely mentioned in scholarship on online abortion storytelling (except in Delay and Sundstrom^56^); that abortion was represented as an embodied process in the ADT stories was therefore an unexpected finding. The emphasis on physicality varied between different abortions – while it was almost absent in accounts of procedural abortions, it was prevalent in medication abortion (MA) narratives, where pregnancy is terminated using abortion pills.^1^ In Poland, as abortion is virtually inaccessible through the healthcare system, most MAs are self-managed, i.e. women terminate pregnancies without medical assistance.^1^ Self-managed MA accounts dominated my dataset. In these narratives, physical symptoms including pain, cramps and bleeding were established as core to the process, but individuals experienced them differently. For some, MA was not very painful and the bleeding was manageable: “It wasn’t terrible at all – just a heavier period”. For most contributors, however, MAs involved significant bleeding and painful cramps, and were sometimes accompanied by additional undesirable physical symptoms, like vomiting or diarrhoea.

*“I won’t sugarcoat it that it didn’t hurt because it hurt very bad. My heating pad and pills didn’t help, I struggled all night. But I feel relief.”*

This abortion was physically challenging – the contributor experienced strong pain, which made her struggle. However, her experience is not mobilised to construct (medication) abortion as harmful to women. Instead, physical symptoms are understood to be a somewhat “normal” part of the MA process – indeed, for others, the absence of pain and bleeding was concerning, as such physical symptoms were understood to signify that the abortion is working. At the same time, physical symptoms were not merely understood as “natural”. Instead, some contributors referred to the way the state and its policies shaped their embodied experiences of abortion:

*“What happened was terrifying … […] 2 hrs of vomiting and severe diarrhoea and terrible stomach pain, during the last half hour I lay exhausted on the bathroom floor and thought that I hate my country for making me go through it like this.”*

In this excerpt, the physical experience is not understood to simply reflect the “nature” of MA. Though it is unclear what abortion experience this contributor would have preferred, implicit in her story is the wish to go through the abortion differently. By stating “I hate my country for making me go through it like this”, the contributor politicises her experience and constructs the Polish state as responsible for what she went through. She problematises the fact that in the absence of appropriate abortion healthcare in Poland, many women have little choice but to self-manage their abortions, sometimes without access to support or effective pain relief.

Simultaneously, some stories presented pain as something that could be managed, and contributors shared practical advice on this:

*“Don’t forget about painkillers. IT DOES NOT HAVE TO HURT. Just make sure you prepare properly.”*

By giving practical advice to other abortion seekers, such testimonies aim to help make the abortion process comfortable for others and reject the notion that abortion must involve suffering. In such stories, pain is understood as a challenge to be tackled, and women use their embodied experiences to give others advice about self-managing the challenging physicality of MA. This advice ranges from information on pain management or pill dosage, to tips on how best to pass time during the abortion.

Contributors further used their embodied lived experience to make sense of the “sensations” of abortion. Specifically, the pain, bleeding and cramps felt during MA were compared to those experienced previously during menstruation or childbirth:

*“Initially the pain was like period pain […]. This lasted until the evening, [the cramps] became more intense. […] In the evening […] when the cramps intensified to the point that I would compare them to the gentle contractions from the early stages of childbirth, the pills began working [… .].”*

This further grounds MA as an embodied process. In such stories, the contributors draw on their lived experiences to highlight the similarities between the embodiment of menstruation, childbirth, and abortion, and therefore these experiences themselves.

Finally, MA was marked as an embodied process through explicit descriptions of the passing of the embryo/fetus^†^ from the body:

*“I just felt something was literally falling out of me. […] It was quite a large tissue, like a clot, well it certainly didn’t look like it does on the banners in front of churches.”*

This extract illustrates that an MA entails a confrontation with the embryo/fetus. Using medical and “neutral” terminology, like “tissue”, “clot”, “fetus” or “embryo”, arguably works to reclaim language co-opted by anti-choice groups, and to challenge representations of abortion as “killing babies” which dominate the Polish mainstream and are mobilised by anti-choice groups.^5,57^ The contributor further explicitly refers to the presence of manipulated images of aborted embryos/fetuses in Polish public spaces and uses her embodied experience to reject those representations (“it certainly didn’t look like it does on the banners in front of churches”).

## Discussion

### Representations of abortion

In the stories shared in the ADT initiative, abortion was represented as a choice and experience imbued with multiple meanings. When discussing abortion decisions, contributors established abortion as a reproductive option women should be able to choose and ascribed all abortion decisions with equal value, which explicitly challenged the idea that there are “good” and “bad” abortions.^27^ However, when talking about their own abortion decisions, contributors drew on a variety of explanations, and some emphasised adverse circumstances and (potential) children’s wellbeing. In doing so, they mobilised “maternal pro-choice” discourses, which rationalise abortion by constructing it as a choice made “not to satisfy [women’s] own desires, but to benefit those around them, particularly their potential children” (p.44).^15^ This positions abortion as a “selfless” (and not “selfish”) choice.^37^ However, other women foregrounded their needs and wants, including not wanting to be a mother. This remains transgressive given the influential, albeit also declining, norms expecting maternal sacrifice from Polish women.^58^ Rejecting motherhood further challenges the assumption that all women naturally desire motherhood, which underlies abortion stigma.^8^ It also rejects the notion that abortions must be “bred from necessity” (p.101) to be acceptable,^59^ or rather, reconstructs the pregnant woman’s desire to remain childfree as a necessity in its own right. Through such testimonies, the idea that women can have abortions because they do not want to be pregnant or have (more) children becomes a “common-sense rationale”.^60,61^ The “selfish aborting woman” stereotype is therefore re-appropriated as women unapologetically centre their needs^15^ – in such stories: any reason for abortion really is good enough.

Representations further portrayed abortion as imbued with diverse emotions and meanings. For some women, abortion was a happy and positive decision. Highlighting “the subject position of the unwillingly pregnant woman for whom abortion is a beneficial and life-enhancing decision” remains transgressive not only in Poland with its longstanding negativity towards abortion, including in pro-choice spaces,^11^ but arguably also across other contexts, particularly those where abortion negativity is also prevalent (see, e.g., Purcell et al^62^). In such testimonies, pregnancy emerges as “an interdependent relationship with women as its subjects and fetuses as part of (not distinct from) their bodies” (p.28).^15^ This understanding of pregnancy challenges mainstream discourses around both abortion and pregnancy, which, by constructing women as mothers to their fetuses and reinforcing ideals of pregnant women’s perpetual “maternal happiness”, position abortion as necessarily tragic.^15^ Instead, by centring the woman’s subjectivity, such stories allow abortion to emerge as a positive and life-giving decision. However, those contributors who wanted or were ambivalent about their pregnancies experienced abortions as challenging due to the complex and contradictory factors shaping their decisions. This highlights that pregnancies can be experienced in complex and diverse ways.^15,63^ However, acknowledging the difficulties and complexities of such abortions does not necessarily reinforce discourses which justify abortion by constructing it as tragic,^19,20,61^ like Polish pro-choice discourses often do.^64^ Rather, representations of abortion as challenging in such stories prompt a more nuanced understanding of abortion, one situated within the specificities of individual pregnancies, and the subjectivities of individual women.^63,65^

Finally, the embodiment of abortion was discussed explicitly in these stories. This arguably constitutes a form of “truth-telling” and moves abortion from the realm of moral choices, instead forcing “a recognition of the lived bodily realities of women’s lives”, simultaneously breaking wider taboos around women’s reproductive health (p.148).^56^ Furthermore, comparing MA to experiences other women will have been through, such as menstruation or childbirth, and explicitly discussing all aspects of its physicality, arguably “intimises” abortion by making it familiar.^30^ This is also true for explicit descriptions of the passing of embryos/fetuses, which further challenges prevalent misconceptions about what aborted embryos/fetuses look like,^16^ and is subversive given that such representations have generally been absent from pro-choice representations of abortion.^66^ Overall, these testimonies “provid[e] pregnant people the resources to navigate their own abortions” (p.19),^67^ which is important as “what abortion actually involves is often socially silenced” (p.586),^9^ and in Poland abortion seekers often encounter misleading information.^1^

### Challenging stigma and deconstructing normative hierarchies

These representations of abortion powerfully challenge abortion stigma on multiple levels. Most obviously, the sole act of sharing an abortion testimony breaks the silence and secrecy around abortion prevalent in Poland, revealing “public secrets”, and challenging abortion exceptionalism by showing that, despite being constructed as such, abortions are not exceptional experiences.^1,8^ The absence of Catholic discourses of abortion, e.g. representations of embryos/fetuses as “babies” or abortion as “murder”, arguably highlights that these discourses, and the stigma they reinforce, are contested and rejected in these stories.^30,57^ The multiplicity of reasons that women gave for their abortions further contributes to destigmatising abortion by portraying it as a “commonplace, essential and life-giving decision for all who choose it” (p.16).^11^ Explicit discussions of the embodiment of abortion also contribute to challenging stigma through strategies of “truth-telling” and “intimisation”, and powerfully break the silence around abortion.^30,56^ Advice-giving, comparing MA with other reproductive experiences and explicitly discussing what it entails (including confrontations with embryos/fetuses) arguably suggests that these testimonies are part of the wider “abortion self-help” which arose after 2016,^28^ as they aim to support women in having abortions,^29^ whilst simultaneously combating stigma by challenging misinformation about abortion^68^ and self-managed MA specifically.^1,69^

Access to safe abortion is recognised as a human right and is core to ensuring good health.^70^ As abortion stigma contributes to upholding restrictive abortion laws (such as those in Poland) and to creating various barriers to access to abortion care,^10^ challenging stigma is necessary to ensure that abortion-seekers can access a full range of reproductive, and human, rights. However, as normative hierarchies of abortion work to stigmatise some abortions, attempts to challenge stigma without deconstructing these hierarchies are, at best, superficial. ^12,24^ Though hierarchies differ between mainstream and pro-choice discourses, both rely on “othering” women who do not fulfil specific norms.^12^ The fact that, in the ADT stories, contributors explicitly ascribed all reasons for abortion with equal value powerfully deconstructs the central premise which normative hierarchies rely on.^12,16^ Further, by portraying multiple, complex and contradictory abortion experiences, and failing to position them on a normative hierarchy, these stories deconstruct normative hierarchies in their multiple manifestations. They therefore also challenge the “narrative” and “thematic” rigidities which characterise some anglophone storytelling projects, and which reproduce abortion stigma by restricting what stories can be told in these spaces.^21,26^ My sample constructs “pregnancy as a subjective and variable condition” (p. 279), one that can be both a negative and positive experience, which results in abortion being represented as something which has multiple meanings.^15,63,65^ As women’s subjectivities are foregrounded, no single meaning can be rendered more “correct” than others, thus deconstructing normative hierarchies.

Despite their subversive potential, the stories highlight some tensions within women’s narratives. Justificatory narratives, e.g. maternal pro-choice discourses, are still used, which highlights their strength and hegemonic status.^15^ Testimonies of challenging abortion experiences also may, “within a hegemonic cultural context that treats abortion as stigmatised or tragic” (p.52), be understood as negative representations.^66^ This concern is valid, however, this narrative diversity and willingness to engage with difficult, contradictory and complex abortion experiences is a strength of these representations.^49^ They challenge abortion stigma and deconstruct normative hierarchies precisely because they offer space for, and value equally, all these experiences. Pro-choice activism which relies on narrow norms around abortion, even when these challenge mainstream norms, and which restricts what can be said, will always marginalise some people, and therefore fail to be liberatory for all.^20^

The contributors’ stories were submitted to ADT’s pro-choice storytelling initiative. When understood as a “pro-choice” strategy, these stories suggest a potential shift and expansion of norms and narratives in some Polish pro-choice spaces. These stories clearly counter the “monolithic and reductive framing of abortion” (p.16), and the associated stigma ascribed to some abortions, characteristic of previous representations, e.g. those during the 2016 protests.^11^ The explicit positivity with which some abortions are portrayed, and the unapologetically “selfish” reasons some women give for their abortions suggest a considerable shift in (pro-choice) norms and narratives in this pro-choice space. ADT further provides space for discussing challenging aspects of abortion or its “graphic” embodiment. This suggests that pro-choice advocacy does not have to rely on normative hierarchies, that these hierarchies can be deconstructed, and that pro-choice activism can grapple with the difficult aspects of abortion. This is not to suggest, however, that hegemonic modes of representing abortion are not influential any more; they are, as was intimated above. Nonetheless, these stories show that representations of abortion that challenge stigma, including its manifestation in normative hierarchies, are present in some Polish pro-choice spaces. Though evidencing the reasons for this is beyond the scope of this study, speculatively these testimonies may be a product of the particular post-2020 moment in Poland. Arguably, the 2020 protests opened up space for understandings of abortion beyond the constraints of apologetic, stigmatising and Catholic discourses,^28^ which may be reflected in these stories. The stories also point to some similarities between my sample, and storytelling in anglophone contexts, indicating that some pro-choice discourses circulate transnationally.^15^ However, existing research on anglophone storytelling highlighted the existence of normative hierarchies in these initiatives,^22,25^ while these hierarchies were deconstructed in my sample. Further research should examine initiatives across different contexts comparatively, and identify reasons for these differences.

### Limitations and future research

This study addressed gaps in the literature pertaining to abortion storytelling in post-2020 Poland, and contributed to scholarship on non-stigmatising representations of abortion.^17^ It pointed to potential changes in Polish pro-choice norms, and added to the literature on stigma by highlighting that explicit discussions of the embodiment of abortion can help destigmatise abortion. However, the findings are specific to this storytelling initiative and cannot be generalised more broadly. It is also important to consider who may be marginalised by these stories.^32^ For example, online storytelling is only available to those with sufficient (digital) literacy,^25,31^ which arguably influences whose stories are present and whose are marginalised within storytelling initiatives like ADT. Nonetheless, this study demonstrated that non-stigmatising, non-negative representations of abortion exist, and these should be explored in other contexts, also comparatively.^17^ More attention should also be given to representations of the embodiment of abortion and its role in challenging stigma in other contexts. In the Polish context, research should explore representations of abortion in other pro-choice initiatives and beyond, to examine whether representations are shifting more broadly.

## Conclusion

Overall, representations of abortion in the ADT storytelling initiative are arguably subversive. The stories analysed here are “uninterested in normalising abortion through oversimplifications, they invited audiences into the tensions, the ambivalences that characterise abortion experiences” (p.57^66^) and force a confrontation with the profoundly embodied aspects of abortion. Through this, they powerfully challenge abortion stigma and its manifestation in normative hierarchies of abortion. Abortion stigma and normative hierarchies have far-reaching consequences, as they influence how people experience, and make sense of, their abortions. At a time when abortion rights and access are being challenged around the world, non-stigmatising representations, such as those in the ADT initiative, can provide an important counter-discourse, and help pave the way for a reality in which abortion is not only accessible and normalised, but perhaps even celebrated. The ADT initiative shows that different representations of abortion are possible, and invites us to reimagine abortion beyond the constraints of stigma.

## Footnotes

* A group helping Polish women obtain abortions in the Czech Republic, see https://ciociaczesia.pl/.https://ciociaczesia.pl/

† I use the term “embryo/fetus” to denote both embryos and fetuses as differences between them are insignificant to my analysis.

## Provenance statement

This article was not commissioned and went through external peer review.

## References

[1] Calkin S. Abortion pills go global: reproductive freedom across borders. Oakland (CA): University of California Press; 2023.

[2] Korolczuk E. Counteracting challenges to gender equality in the era of anti-gender campaigns: competing gender knowledges and affective solidarity. Soc Polit. 2020;27(4):694–717. doi:10.1093/sp/jxaa021

[3] European Parliament. Polish de facto ban on abortion puts women’s lives at risk, says Parliament; 2020. Available at: https://www.europarl.europa.eu/news/en/press-room/20201120IPR92132/polish-de-facto-ban-on-abortion-puts-women-s-lives-at-risk-says-parliament [Accessed: 20.11.2023].

[4] Federa. Dwa lata od wejścia w życie wyroku TK: pełen dostęp do aborcji potrzebny natychmiast; 2023. Available at: https://federa.org.pl/dwa-lata-po-wyroku-tk/ [Accessed: 17.03.2023].

[5] Graff A. Świat bez kobiet. Płeć w polskim życiu publicznym. Warszawa: Wydawnictwo Marginesy; 2021.

[6] Mishtal J. The politics of morality: the church, the state, and reproductive rights in postsocialist Poland. Athens (OH): Ohio University Press; 2015.

[7] Chełstowska A. Stigmatisation and commercialisation of abortion services in Poland: turning sin into gold. Reprod Health Matters. 2011;19(37):98–106. doi:10.1016/S0968-8080(11)37548-9 21555090

[8] Kumar A, Hessini L, Mitchell EMH. Conceptualising abortion stigma. Cult Health Sex. 2009;11(6):625–639. doi:10.1080/13691050902842741 19437175

[9] Purcell C. The sociology of women’s abortion experiences: recent research and future directions. Sociol Compass. 2015;9(7):585–596. doi:10.1111/soc4.12275

[10] Millar E. Abortion stigma as a social process. Wom Stud Int Forum. 2020;78:1–7.

[11] Cullen P, Korolczuk E. Challenging abortion stigma: framing abortion in Ireland and Poland. Sex Reprod Health Matt. 2019;27(3):6–19. doi:10.1080/26410397.2019.1686197 PMC7887965 31823693

[12] Leask M. An exceptional choice? How young New Zealand women talk about abortion. Aust Femin Stud. 2015;30(84):179–198. doi:10.1080/08164649.2015.1046305

[13] Smith BEY, Bartz D, Goldberg AB, et al. ‘Without any indication’: stigma and a hidden curriculum within medical students’ discussion of elective abortion. Soc Sci Med. 2018;214:26–34. doi:10.1016/j.socscimed.2018.07.014 30138842

[14] Kimport K, Weitz TA, Freedman L. The stratified legitimacy of abortions. J Health Soc Behav. 2016;57(4):503–516. doi:10.1177/0022146516669970 27856971

[15] Millar E. Happy abortions. Our bodies in the era of choice. London: Zed Books Ltd; 2017.

[16] Becker A, Hann LR. ‘It makes it more real’: examining ambiguous fetal meanings in abortion care. Soc Sci Med. 2021;272:1–9. 10.1016/j.socscimed.2021.113736 33588202

[17] Baird B, Millar E. More than stigma: interrogating counter narratives of abortion. Sexualities. 2019;22(7–8):1110–1126. doi:10.1177/1363460718782966

[18] Nickerson A, Manski R, Dennis A. A qualitative investigation of low-income abortion clients’ attitudes toward public funding for abortion. Wom Health. 2014;54(7):672–686. doi:10.1080/03630242.2014.919984 25068780

[19] McKinney C. A good abortion is a tragic abortion: fit motherhood and disability stigma. Hypatia. 2019;34(2):266–285. doi:10.1111/hypa.12461

[20] Ludlow J. The things we cannot say: witnessing the traumatization of abortion in the United States. Wom Stud Quart. 2008;36(1–2):28–41. doi:10.1353/wsq.0.0057

[21] Ralph D. First-person abortion story-sharing as key pro-choice strategy. In: Abortion and Ireland. Cham: Springer International Publishing; 2020. p. 39–68.

[22] Willis R. Whose story is it anyway? Hashtag campaigns and digital abortion storytelling. In: T Vine, S Richards, editors. Stories, storytellers, and storytelling. Cham: Springer International Publishing; 2022. p. 145–172.

[23] Siegel DP. Wanting a ‘feminist abortion experience’: emotion work, collective identity, and pro-choice discourse. Sociolog Forum. 2021;36(2):471–490. doi:10.1111/socf.12688

[24] Cockrill K, Nack A. “I’m not that type of person”: managing the stigma of having an abortion. Deviant Behav. 2013;34(12):973–990. doi:10.1080/01639625.2013.800423

[25] Ralph D. “Between a whisper and a shout”: repealing the eighth and pro-choice Irish women’s abortion testimonies. Gender Place Cult. 2022;29(2):201–221. doi:10.1080/0966369X.2020.1860911

[26] Allen M. Narrative diversity and sympathetic abortion: what online storytelling reveals about the prescribed norms of the mainstream movements. Symb Interact. 2015;38(1):42–63. doi:10.1002/symb.135

[27] Weitz TA. Rethinking the mantra that abortion should be “safe, legal, and rare”. In: CE Joffe, JA Reich, editors. Reproduction and society. 1st ed. [Online]. New York, NY: Routledge; 2015. p. 67–75.

[28] Graff A, Korolczuk E. Kto się boi gender? Prawica, populizm i feministyczne strategie oporu. Translated from English by M. Sutowski. Warszawa: Wydawnictwo Krytyki Politycznej; 2022.

[29] Dzwonkowska-Godula K. Samopomoc i samoobsługa aborcyjna w czasach niepewności. Kultura i Społeczeństwo. 2022;66(1):25–52. doi:10.35757/KiS.2022.66.1.2

[30] Chełstowska A, Ignaciuk A. Criminalization, medicalization, and stigmatization: genealogies of abortion activism in Poland. Signs J Women Cult Soc. 2023;48(2):423–453. doi:10.1086/722897

[31] Belfrage M, Didier E, Vázquez-Quesada L. Voicing abortion experiences to reduce stigma: lessons from an online storytelling platform in Mexico. Wom Reprod Health. 2022;9(3):203–217. doi:10.1080/23293691.2021.2016145

[32] Mishler K. “It’s most peculiar that this particular story doesn’t get told”: a reproductive-justice analysis of storytelling in the repeal campaign in Ireland, 2012–18. Éire-Ireland. 2021;56(3–4):80–103. doi:10.1353/eir.2021.0016

[33] Braun V, Clarke V. Successful qualitative research: a practical guide for beginners. First ed. London: SAGE LTD; 2013.

[34] Hall S. The work of representation. In: S Hall, editors. Representation: cultural representations and signifying practices. London: SAGE; 1997. p. 13–64.

[35] ADT (Aborcyjny Dream Team). “Miałam aborcję” – wasze historie; n.d. -a. Available at: https://aborcyjnydreamteam.pl/mialam-aborcje/ [Accessed: 20.03.2023].

[36] ADT (Aborcyjny Dream Team). O ADT; n.d. -b. Available at: https://aborcyjnydreamteam.pl/o-nas/ [Accessed: 26.09.2023].

[37] Becker A. “My abortion made me a good mom”: an analysis of the use of motherhood identity to dispel abortion stigma. Reproduction, Health and Medicine. 2020;20:219–240.

[38] Byrne D. A worked example of Braun and Clarke’s approach to reflexive thematic analysis. Qual Quant. 2022;56(3):1391–1412. doi:10.1007/s11135-021-01182-y

[39] Braun V, Clarke V. Reflecting on reflexive thematic analysis. Qual Res Sport Exerc Health. 2019;11(4):589–597. doi:10.1080/2159676X.2019.1628806

[40] Braun V, Clarke V. One size fits all? What counts as quality practice in (reflexive) thematic analysis? Qual Res Psychol. 2021;18(3):328–352. doi:10.1080/14780887.2020.1769238

[41] Braun V, Clarke V. Using thematic analysis in psychology. Qual Res Psychol. 2006;3(2):77–101. doi:10.1191/1478088706qp063oa

[42] Janetzko D. Nonreactive data collection online. In: N Fielding, RM Lee, G Blank, editors. The SAGE handbook of online research methods. London: SAGE Publications Ltd; 2017. p. 76–91.

[43] Abfalter D, Mueller-Seeger J, Raich M. Translation decisions in qualitative research: a systematic framework. Int J Soc Res Methodol. 2021;24(4):469–486. doi:10.1080/13645579.2020.1805549

[44] Temple B, Young A. Qualitative research and translation dilemmas. Qual Res. 2004;4(2):161–178. doi:10.1177/1468794104044430

[45] Reich JA. Power, positionality, and the ethic of care in qualitative research. Qual Sociol. 2021;44(4):575–581. doi:10.1007/s11133-021-09500-4

[46] Lee RM, Renzetti CM. The problems of researching sensitive topics: an overview and introduction. In: CM Renzetti, RM Lee, editors. Researching sensitive topics. Newbury Park: Sage; 1993. p. 3–13.

[47] Gillies V, Alldred P. The ethics of intention: research as a political tool. In: M Mauthner, M Birch, J Jessop, T Miller, editors. Ethics in qualitative research. London: Sage; 2012. p. 32–52.

[48] Doucet A, Mauthner NS. Knowing responsibly: ethics, feminist epistemologies and methodologies. In: T Miller, M Birch, M Mauthner, J Jessop, editors. Ethics in qualitative research. London: Sage; 2012. p. 122–139.

[49] Kimport K. (Mis)understanding abortion regret. Symb Interact. 2012;35(2):105–122. doi:10.1002/symb.11

[50] Mavuso JM-JJ, Chiweshe MT, Macleod CI. ‘Choice’ in women’s abortion decision-making narratives: introducing a supportability approach. Psychol Soc. 2020;59:20–40. doi:10.57157/pins2020Vol59iss1a5605

[51] Amuchastegui A. Body and embodiment in the experience of abortion for Mexican women: the sexual body, the fertile body, and the body of abortion. Gender Sex Femin. 2013;1(1):1–17. doi:10.3998/gsf.12220332.0001.101

[52] McCulloch H, Perro D, Taghinejadi N, et al. Expectations and experiences of pain during medical abortion at home: a secondary, mixed-methods analysis of a patient survey in England and Wales. BMJ Sex Reprod Health. 2025;51(2):137–143. doi:10.1136/bmjsrh-2024-202533 39694584

[53] Purcell C, Brown A, Melville C, et al. Women’s embodied experiences of second trimester medical abortion. Fem Psychol. 2017;27(2):163–185. doi:10.1177/0959353517692606 28546655 PMC5431358

[54] Purcell C, Newton VL, Bloomer F, et al. Foregrounding pain in self-managed early medication abortion: a qualitative study. BMJ Sex Reprod Health. 2025;51(1):3–8. doi:10.1136/bmjsrh-2023-202198 38429082

[55] Roseth I, Lyberg AM, Sommerseth E, et al. “Out of this world”: Norwegian women’s experiences of medical abortion pain. J Multidiscip Healthc. 2023;16:889–898. doi:10.2147/JMDH.S399209 37038454 PMC10082597

[56] Delay C, Sundstrom B. ‘In her shoes’ and in her words: voices, silences, and bodies in Irish women’s abortion narratives. Frontiers (Boulder). 2022;43(2):139–168.

[57] Szelewa D. Killing “unborn children”? The Catholic Church and abortion law in Poland since 1989. Soc Leg Stud. 2016;25(6):741–764. doi:10.1177/0964663916668247

[58] Imbierowicz A. The Polish mother on the defensive? The transformation of the myth and its impact on the motherhood of Polish women. J Educ Cult Soc. 2012;2012(1):140–153. doi:10.15503/jecs20121.140.153

[59] Swafford S. To be a (m)other: a feminist performative autoethnography of abortion. Cult Stud Crit Methodol. 2020;20(2):95–103. doi:10.1177/1532708619878743

[60] Beynon-Jones SM. Untroubling abortion: a discourse analysis of women’s accounts. Fem Psychol. 2017;27(2):225–242. doi:10.1177/0959353517696515 28546656 PMC5431366

[61] Combellick SL. “My baby went straight to heaven”: morality work in abortion online storytelling. Soc Probl. 2021;70(1):87–103. doi:10.1093/socpro/spab033

[62] Purcell C, Maxwell K, Bloomer F, et al. Toward normalising abortion: findings from a qualitative secondary analysis study. Cult Health Sex. 2020;22(12):1349–1364. doi:10.1080/13691058.2019.1679395 31933421 PMC7611965

[63] Lupton D. The social worlds of the unborn. Basingstoke: Palgrave Macmillan; 2013.

[64] Pamuła N. “Czy adoptowałeś już niepełnosprawne dziecko?”: przyczynek do analizy niepełnosprawności w dyskursie pro-choice w Polsce. Teksty drugie. 2020;2(2):86–103. doi:10.18318/td.2020.2.6

[65] Leach BR. Abjection and mourning in the struggle over fetal remains. Contemp Polit Theory. 2021;20(1):141–164. doi:10.1057/s41296-020-00400-w

[66] Ludlow J. It’s a boy!borted fetal bodies, graphic abortion, and the option to look. In: Hurst RAJ, editors. Representing abortion. Abingdon, Oxon: Routledge; 2021. p. 49–60.

[67] Siegel DP. Medicalization and naturalization: understanding abortion as a naturecultural phenomenon. Catal Femin Theory Technosci. 2020;6(2):1–26. doi:10.28968/cftt.v6i2.34030

[68] Norris A, Bessett D, Steinberg JR, et al. Abortion stigma: a reconceptualization of constituents, causes, and consequences. Wom Health Iss. 2011;21(3):S49–S54. doi:10.1016/j.whi.2011.02.010 21530840

[69] Broussard K. The changing landscape of abortion care: embodied experiences of structural stigma in the Republic of Ireland and Northern Ireland. Soc Sci Med. 2020;245:1–9. 10.1016/j.socscimed.2019.112686 PMC6930969 31775107

[70] World Health Organisation. Abortion care guidelines; 2022. Available at: https://www.who.int/publications/i/item/9789240039483 [Accessed: 20.04.2025].

